# Knowledge and preventive practice to COVID-19 among household heads in Enugu metropolis, South-East Nigeria

**DOI:** 10.11604/pamj.2020.37.63.23986

**Published:** 2020-09-16

**Authors:** Elizabeth Uzoamaka Nwonwu, Edmund Ndudi Ossai, Chukwuma David Umeokonkwo, Ituma Bernard Ituma

**Affiliations:** 1 Department of Community Medicine, College of Health Sciences Ebonyi State University, Abakaliki, Nigeria,; 2 Department of Community Medicine, Alex Ekwueme Federal University Teaching Hospital, Abakaliki, Ebonyi State, Nigeria,; 3 Nigeria Field Epidemiology Training Program, Abuja, Nigeria

**Keywords:** COVID-19, knowledge, preventive practices, non-pharmaceutical interventions, Enugu metropolis

## Abstract

**Introduction:**

the coronavirus disease COVID-19 pandemic has affected the way we live. The use of non-pharmaceutical interventions (NPI) has been reported to be effective in controlling similar respiratory diseases outbreak in the past and is being used as one of the mainstays of control of the pandemic. We therefore assessed the knowledge and practice of preventive measures against COVID-19 among adults in Enugu metropolis during the outbreak in the State.

**Methods:**

we conducted a descriptive cross-sectional study among 320 adults who were recruited through a multistage sampling technique. We used semi-structured interviewer-administered questionnaire to collect information on the knowledge and preventive practices against COVID-19. The data were analyzed using the IBM-SPSS version 25.

**Results:**

the mean age of the participants was 41.6±12.5 years. There were 168 (52.5%) male, 69.7% had attained tertiary education and 57.8% were self-employed. Overall, 256 (80.0%) of the respondents had good knowledge about COVID-19. Only 133 (41.6%) had good practice of preventive measures among respondents. The more commonly practiced NPIs among the respondents were use of alcohol hand sanitizer (86.6%), physical distancing (85.6%), washing of hands with soap and water (81.6%) and disinfecting frequently touched surfaces (80.9%). The NPIs the respondents practiced poorly were use of face mask (33.8%) and avoidance of crowded areas (47.2%). Having good knowledge (aOR: 3.2; 95% CI: 1.65 - 6.05) and attaining secondary education or less (aOR: 2; 95%CI: 10-3.13) were the only predictors of good practice of preventive measures.

**Conclusion:**

the highly educated segment of the population needs to be targeted with appropriate messages to improve their adoption of the right preventive measures against COVID-19.

## Introduction

The coronavirus disease (COVID-19) is caused by a new strain of coronavirus (SARS-CoV-2) that has not been previously identified in humans. It was first reported to World Health Organization on December 31^st^, 2019 after it was isolated in a cluster of patients in Wuhan China. COVID-19 has the same veiled RNA structure resembling SARS-CoV and MERS-CoV but it is more infectious [1]. The basic idea about COVID-19 infection is that it spreads through the direct mucus contact with the breath/ingestion/salivary droplet of an infected person that can live on hands, objects or surfaces [1,2]. The outbreak was declared a public health emergency of international concern on January 30^th^, 2020 by WHO and called for collaborative efforts of all countries to prevent the rapid spread of COVID-19 [3]. The ongoing COVID-19 pandemic has spread rapidly and by end of May 2020, the virus has reached over 215 countries, areas and territories resulting in more than 6 million confirmed cases and 370, 000 deaths worldwide including Nigeria.

Nigeria Centre for Disease Control announced the first case of coronavirus disease in Nigeria on February 27, 2020 and since then many confirmed cases have been reported in many States across the country. As at the end of May 2020, over 10, 162 cases have been confirmed, 3007 cases have been discharged and 287 deaths have been recorded in 35 States and the Federal Capital Territory. In Enugu State 18 cases have been confirmed, 12 discharged and no death. To reduce the continued spread of the coronavirus disease and its associated mortality, World Health Organization has recommended series of preventive measures including regular hand washing with water and soap, social distancing, covering nose and mouth while coughing and avoid touching eyes, nose and mouth. Implementing personal hygiene and public health behaviors such as hand washing and social distancing are necessary to curb the spread of coronavirus, but it will be challenging to practice these in many cities and rural areas in developing countries. Without sustained bans on large gatherings (including specific cultural and faith practices such as mass prayer gatherings, large weddings and funerals) these may create super-spreading events that accelerate transmission [4]. This situation may be compounded by the spread of COVID-19 misinformation including unsupported treatments or promotion of ineffective preventive behaviors [5].

In Nigeria, preventive measures have been adopted to prevent further spread of the virus in the country. The government of Nigeria has also engaged in media campaigns to disseminate information on preventive measures to the general public. However, the level of knowledge and preventive practices against COVID-19 infection among adults in Enugu are yet to be evaluated. Therefore, this study aimed to determine the knowledge and preventive practices against coronavirus infection among adults in Enugu metropolis, Nigeria.

## Methods

**Study setting**: Enugu is the capital of Enugu State which is one of the five States in the south-east geo-political zone in Nigeria. The metropolis is made up of three local government areas namely Enugu North, Enugu South and Enugu East local government areas. Its population according to the 2006 national population census was 722,664 people with a growth rate of 3.05% [6]. Its inhabitants are mainly of Igbo ethnic nationality and are predominantly Christians. The occupation of the people includes civil service, trading, artisanship and farming.

**Study design and population**: this was a descriptive cross-sectional study on heads of households in Enugu metropolis, Enugu state, Nigeria. A household is a group of people who live together and feed from the same pot. The head of household is the individual responsible for leadership and financial decisions in the household. We estimated the minimum sample size using the formula for single proportions.

$$n=\frac{Z^{2}P(1-P)}{d^{2}}$$

A sample size of 320 respondents was estimated based on a type 1 error (α) of 0.05, tolerable margin of error of 0.05 and a proportion of 83.9%, representing the proportion of respondents that had good knowledge of COVID-19 in a study among Nigerians [7].

**Sampling technique and study instrument**: a multistage sampling technique was used to recruit respondents for the study. In the first stage, a simple random sampling technique of balloting was used to select one out of the three local government areas in the metropolis. In the second stage, a simple random sampling technique of balloting was used to select two out of four districts in the selected local government area. In the third stage, a simple random sampling technique of balloting was used to select two communities each from the list of communities in the two districts that are located in the urban section of the selected local government area. In the fourth stage, a systematic random sampling technique was used to select houses in the selected communities. House numbering was done in each of the selected communities and that served as the sampling frame. A total of 80 respondents were allocated to each of the four communities. The sampling interval for each of the selected communities was determined by dividing the sampling frame by the sample size of 80. The sequence that the houses were selected was based on the numbers allocated to the houses. The index house was selected using a simple random sampling technique of balloting. In the fifth stage, a list of households in each of the selected houses where there were more than one household was made and one household was selected using a simple random sampling technique of balloting. The head of each of the selected household was included in the study. A pretested semi-structured questionnaire developed by the researchers was used for the study. The questionnaire had three sections. The first section had information on socio-demographic characteristics, second section assessed the knowledge on COVID-19 and the third section assessed the practice of the preventive measures on COVID-19. The questionnaire was administered to the respondents by trained research assistants.

**Outcome measure**: outcome measure included Good knowledge and Good preventive practices against COVID-19. Knowledge of COVID-19 was assessed using 22 variables. A correct response to each of the variables was awarded a score of one while an incorrect answer was scored zero. Respondents who scored ≥70% of the 22 variables were regarded as having good knowledge of COVID-19. Preventive practices against COVID-19 was assessed using ten variables. A correct response to each of the variables was given a score of one while an incorrect answer was scored zero. Respondents who scored ≥70% of the ten variables were designated as having good preventive practices against COVID-19. The socio-economic status index was developed using Principal Component Analysis, (PCA) in STATA statistical software version 12. The input to the PCA included information on estimated household monthly income and ownership of ten household items that included radio, television, cable television, generator, gas cooker, refrigerator, electric iron, car, air-conditioner and washing machine. For calculation of distribution cut points, quartiles, (Q) were used. Each respondent was assigned the wealth index score of the household. The quartiles were Q1 = poorest, Q2= the very poor, Q3= the poor and Q4= least poor. The first two quartiles, Q1 and Q2 were grouped as low socio-economic class while Q3 and Q4 were categorized as high socio-economic class.

**Data management**: data entry and analysis were done using IBM Statistical Package for Social Sciences statistical software version 25. Frequency tables and cross-tabulations were generated. Chi square test was used to assess the relationship between the good knowledge and sociodemographic characteristics. Multivariate analysis using binary logistic regression was used to determine predictors of good knowledge and good preventive practices against COVID-19. Variables that had a p value of <0.2 on bivariate analysis were entered into the logistic regression model to determine the predictors of good knowledge and good preventive practices against COVID-19. Result of regression analysis was reported using adjusted odds ratio and 95% confidence interval at 5% level of significance.

**Ethical considerations**: ethical approval for the study was obtained from the Research and Ethics Committee of Enugu State Ministry of Health, Enugu state, Nigeria. We obtained a written informed consent from the respondents before questionnaire administration. The participants were assured by the researchers that participation in the study was voluntary and that information obtained for the study will be treated anonymously and confidentially.

## Results

The mean age of the respondents was 41.6 ± 12.5 years. There were 168 (52.5%) male, 74.7% of the respondents were married, 20.5% (65) had more than four children, 94.1% were Christians, 69.7% had attained tertiary education and 57.8% were self-employed (Table 1). All the respondents were aware of COVID-19 pandemic and the major source of their information on COVID-19 were television 94.7%, friends 81.9%, social media 80.3%, radio 72.2% and family members 67.2%. The least common sources of information for them were market 19.4% and town criers (4.7%). The most common symptoms of COVID-19 the respondents were aware of were fever (85.0%), sneezing (79.1%), dry cough (76.9%) and difficulty in breathing (63.7%). Overall, 80.0% (256) of the respondents had good knowledge about COVID-19, none of the socio-demographic characteristics examined was found to be associated with having good knowledge of the COVID-19 (Table 2).

**Table 1:** socio-demographic characteristics of respondents

Variable	Frequency (n=320)	Percent (%)
**Age (years)**		
Mean SD	41.6±12.5	
<35 years	111	34.7
35-44 years	100	31.3
≥45 years	109	34.1
**Gender**		
Male	168	52.5
Female	152	47.5
**Number of children**		
None	72	22.5
1-4 children	183	57.2
≥5 children	65	20.3
**Marital status**		
Never married	69	21.6
Married	239	74.7
Divorced	7	2.2
Separated	5	1.6
**Religion**		
Christianity	301	94.1
Islam	17	5.3
Traditional religion	2	0.6
**Education**		
No formal	13	4.1
Primary	9	2.8
Secondary	75	23.4
Tertiary	223	69.7
**Employment status**		
Unemployed	11	3.4
Self-employed	185	57.8
Salaried employment	124	38.8
**Socio-economic class**		
Low socio-economic class	164	51.2
High socio-economic class	156	48.8

**Table 2:** factors affecting good knowledge of COVID-19 among the respondents

Variable	Good Knowledge N (%)	Poor knowledge N (%)	P value
**Age (years)**			
<35	84 (75.7)	27 (24.3)	0.270
35-44	80 (80.0)	20 (20.0)	
≥45	92 (84.4)	17 (15.6)	
**Gender**			
Male	136 (81.0)	32 (19.0)	0.654
Female	120 (78.9)	32 (21.1)	
**Number of children**			
None	52 (72.2)	20 (27.8)	0.170
1-4 children	150 (82.0)	33 (18.0)	
≥5 children	54 (83.1)	11 (16.9)	
**Marital status**			
Married	196 (82.0)	43 (18.0)	0.123
Others^**^	60 (74.1)	21 (25.9)	
**Education**			
Tertiary	179 (80.3)	44 (19.7	0.855
Secondary and less	77 (79.4)	20 (20.6)	
**Employment status**			
Unemployed	9 (81.8)	2 (18.2)	0.852
Self-employed	146 78.9)	39 (21.1)	
Salaried employment	101 (81.5)	23 (18.5)	
**Socio-economic class**			
Low socio-economic class	128 (78.0)	36 (22.0)	0.371
High socio-economic class	128 (82.1)	28 (17.9)	

** Never married, separated, divorced

The use of preventive measures (non-pharmaceutical interventions NPI) among respondents was observed to be generally poor among the respondents. Only 41.6% (133) had good practice of preventive measures among respondents (Table 3). The most common NPI practiced among the respondents were use of alcohol hand sanitizer (86.6%), physical distancing (85.6%), washing of hands with soap and water (81.6%) and disinfecting frequently touched surfaces (80.9%). The NPIs the respondents practiced poorly were use of face mask (33.8%) and avoidance of crowded areas (47.2%, Table 3) Education, employment status and having good knowledge of COVID-19 were found to be associated with good practice of preventive measures against COVID-19 among the participants (Table 4), however when modelled to remove the possible effect of confounder, having good knowledge (aOR: 3.2; 95% CI: 1.65 - 6.05) and attaining secondary education or less (aOR: 2; 95%CI: 1.10-3.13) were the only predictors of good practice of preventive measures. Those who had good knowledge of the disease were 3.2 times higher odds of having good practice of the preventive measures compared to those with poor knowledge of the disease. Those who have secondary education or less had twice higher odds of practicing good preventive measures compared to those that had attained tertiary education (Table 5).

**Table 3:** practices of preventive measures against COVID-19 among the respondents

Variable	Frequency	Percent (%)
**In the last two weeks**		
**Have gone to a crowed place**		
Yes	151	47.2
No	169	52.8
**Have travelled out of state for a social function**		
Yes	101	31.6
No	219	68.4
**Have worn a face mask when leaving home**		
Yes	108	33.8
No	212	66.2
**Wash hands with soap and water regularly**		
Yes	261	81.6
No	59	18.4
**Use personal alcohol-based sanitizer**		
Yes	277	86.6
No	43	13.4
**Shake hands with other people regularly**		
Yes	182	56.9
No	138	43.1
**Touch face/nose/eyes with hands regularly**		
Yes	246	76.9
No	74	23.1
**Clean/disinfect frequently touched surfaces**		
Yes	259	80.9
No	61	19.1
**Stay indoors from time to time to prevent infection**		
Yes	252	78.8
No	68	21.3
**Maintain at least one meter from others in public places**		
Yes	274	85.6
No	46	14.4
**Preventive practices against COVID-19**		
Good	133	41.6
Poor	187	58.4

**Table 4:** factors affecting good preventive practices against COVID-19

Variable	Good preventive practice N (%)	Poor preventive practice N (%)	p value^*^
**Age (year)**			
<35	44 (39.6)	67 (60.4)	0.796
35-44	41 (41.0)	59 (59.0)	
≥45	48 (44.0)	61 (56.0)	
**Gender**			
Male	65 (38.7)	103 (61.3)	0.273
Female	68 (44.7)	84 (55.3)	
**Number of children**			
None	25 (34.7)	47 (65.3)	0.380
1-4 children	81 (44.3)	102 (55.7)	
≥5 children	27 (41.5)	38 (58.5)	
**Marital status**			
Married	103 (43.1)	136 (56.9)	0.339
Others^*^	30 (37.0)	51 (63.0)	
**Education**			
Tertiary	86 (38.6)	137 (61.4)	0.099
Secondary and less	47 (48.5)	50 (51.5)	
**Employment status**			
Unemployed	7 (63.6)	4 (36.4)	0.141
Self-employed	70 (37.8)	115 (62.2)	
Salaried employment	56 (45.2)	68 (54.8)	
**Socio-economic class**			
Low	69 (42.1)	95 (57.9)	0.849
High	64 (41.0)	92 (59.0)	
**Knowledge of COVID-19**			
Good	119 (46.5)	137 (53.5)	<0.001
Poor	14 (21.9)	50 (78.1)	

* P valued based on Chi Square statistics ^**^Never married, separated, divorced

**Table 5:** predictors of good preventive practices against COVID-19

Variable	AOR (95% CI)	P value
Education		
Secondary and less	2 (1.10-3.13)	0.021
Tertiary	1	
**Employment status**		
Unemployed	2.2 (0.57-8.09)	0.257
Self-employed	0.6 (0.37-1.02)	0.060
Salaried employment	1	
**Knowledge of COVID-19**		
Good	3.2 (1.65-6.05)	0.001
Poor	1	

## Discussion

We found that the heads of households in Enugu metropolis were all aware of the COVID-19 disease. This is good and signifies that the awareness creation effort of the government and the various public health agencies had yielded some result. Awareness and good perception have been reported as a good predictor of proper preventive measures to infectious disease [8]. The main sources of the information about the diseases were found to be the news media and social media. The finding was similar to an earlier study which reported that high proportion of the respondents sourced their information from social media [9,10]. The use of traditional means of information dissemination was not popular among this population. This could be due to the high proportion of well-educated people among the respondents and the metropolitan nature of the study population.

We observed that a high proportion of the respondents had good knowledge about the COVID-19. However, this knowledge was not found to be associated with any of the sociodemographic characteristics assessed. Age and economic status have however been reported to be a predictor of good knowledge among some health workers [10,11]. This showed a good penetration of risk communication messaging across the different layers of the community. The level of knowledge reported in this study was higher than those earlier reported among health workers in China [9]. We observed that the practice of preventive measures against COVID-19 was generally poor among the respondents. This is despite good knowledge of the disease. It is however important to note that the practices of the preventive measures varied from one measure to another. The use of the preventive measures which are also generally called non pharmaceutical interventions has been noted to help in limiting the transmission of the virus from one person to another [12]. Its effectiveness depends on the right use of a cocktail of the measures. One cannot be fully protected if one practices the use of face mask alone and do not wash hands regularly. Hence despite the good practice observed among some popular measures, the use of the combination of measures, which ensures better preventive effectiveness, was rather poor among the populace. This could be due to incomplete risk communication or other factors such as cost of procuring the materials or its availability. It could also be due to personal preferences or level of risk perception to being infected.

Education and good knowledge of the COVID-19 were the only factors that predicted the good practice of preventive measures against the disease. Having lower educational attainment was associated with greater odds of practicing the preventive measures against COVID-19. This could be as a result of the more acceptance among this segment of the population. The more educated segment of the population were less accepting of the preventive measures probably because they feel less at risk of contracting the diseases. They are less likely to live in crowded areas, more likely to have their own personal means of transportation and feel less exposed to the risk of the exposure to the virus. Having good knowledge about the disease is known to be associated with good preventive practices. Those that know about the diseases, its effects and the mode of transmission are more likely to better prevent themselves from being infected. Efforts should be targeted to improving the level of knowledge in the community about COVID-19 as this will have multiplier effects on the preventive practices against the disease.

## Conclusion

The national public health institute, the Nigeria Center for Disease Control and other national and subnational public health agencies need to improve on the risk communication messaging on the use of the NPIs to help improve the acceptance and practice of these NPIs. The highly educated segment of the population needs to be target with appropriate messages to improve their adoption of the right preventive measures against COVID-19. Effort at educating the whole population about the COVID-19 disease should be encouraged as this has a multiplier effect on also improving their use of preventive practices.

### What is known about this topic

- Effective use of the non-pharmaceutical interventions has been known to limit the spread of respiratory diseases spread by droplet;
- Good knowledge of the disease is associated with practice of preventive measures to protect against infection.

### What this study adds

- The knowledge of COVID-19 is high among the adults in Enugu metropolis;
- However, the practice of preventive measures is low, and this might have implications for the control of the spread of the disease in the metropolis.
